# Reclassification of chronic kidney disease in the elderly: integrating age-adjusted GFR and frailty assessment in a regional Italian population (Abruzzo)

**DOI:** 10.1007/s11255-025-04698-6

**Published:** 2025-07-31

**Authors:** Fabrizio Cristiano, Carlos Guido Musso

**Affiliations:** 1 Department of Neuroscience, Imaging and Clinical Sciences, Gabriele d’Annunzio University of Chieti and Pescara, 66100 Chieti, Italy; 2 ASL 2 Lanciano Vasto Chieti, Nephrology and Dialysis Unit, Ortona Hospital, 66026 Ortona, CH Italy; 3 Nephrology Department, Hospital Italiano of Buenos Aires, Buenos Aires, Argentina

**Keywords:** Chronic kidney disease, Aging kidney, Frailty, GFR estimation, Keller formula, CKD-EPI

## Abstract

**Background:**

Chronic kidney disease (CKD) is increasingly prevalent among the elderly, yet current diagnostic criteria often fail to distinguish between true CKD and age-related physiological decline in glomerular filtration rate (GFR). This overestimation can lead to overdiagnosis, overtreatment, and psychological distress. Frailty, a common condition in older adults, further complicates the clinical picture. This study aimed to reclassify CKD in the elderly by integrating age-adjusted GFR estimation and frailty assessment in a regional Italian population.

**Methods:**

Retrospective, population-based study involving 325,622 individuals aged ≥ 65 years residing in the Abruzzo region. Data included serum creatinine, eGFR (CKD-EPI), urine tests, renal imaging, and frailty measures using the Clinical Frailty Scale (CFS) and Fried Frailty Criteria (FFC). The Keller formula (GFR = 130 − age) was applied to distinguish between physiological renal aging and pathological CKD. Patients were categorized into four groups: Robust CKD, Senescent Nephropathy, Robust Aged Kidney, and Frailty Aged Kidney.

**Results:**

Of the 58,611 elderly patients classified with CKD stages G3–G5 based on CKD-EPI, only 27.9% (65–74 years), 56.0% (75–84 years), and 54.0% (≥ 85 years) had eGFR values below age-adjusted expectations. More than 40% of patients met criteria for renal senescence rather than true CKD. Over 50% of CKD patients fell into frail phenotypes (Senescent Nephropathy or Frailty Aged Kidney), emphasizing the need for a multidimensional clinical approach.

**Conclusions:**

Reclassifying CKD using age-adjusted GFR and frailty assessment improves diagnostic accuracy in the elderly, preventing misdiagnosis and guiding personalized care. This approach supports a shift from static staging to a more nuanced, patient-centered nephrological model that integrates renal physiology and geriatric assessment.

## Introduction

Aging together with low birth rate is a demographic problem for Europe. In Italy over-65 s are 21.4% compared to the European average of 18.5%. In Italy, the regions with highest rate of over-65 s are Liguria (28.2%) and Friuli Venezia Giulia (26%), while the lowest rate is Campania (17.9%); Abruzzo has one of the highest rates of over-65 s in the country at 25.6% [1]. The definition of the old individual related to Observational Report [2] analyses three groups: young-old (65–74 years), old (75–84 years) and very old (over 85 years). In Italy, life expectancy is 83.3 years, of which 81 years for men and 85.5 years for women [3], leading to an increase in the incidence of chronic diseases such as chronic kidney disease (CKD). The incidence of CKD in the old is due to incorrect interpretation of the results obtained by applying the commonly used formulas for eGFR with diagnostic errors. CKD, according to NKF/KDOQI criteria is diagnosed when eGFR < 60 ml/min/1.73m^2^ or signs of renal damage such as albuminuria for a period of more than three months [4]. CKD affects more than 10% of the world’s population, is associated with cardiovascular problems and the prevalence is higher in older patients related to glomerular filtration rate (eGFR) and albuminuria (A) [5]. The National Health and Nutrition Examination Survey (NHANES) has examined the prevalence of CKD among adults in the United States. According to data collected between 2017 and March 2020, 35.5 million of the population adults (14%) have CKD particulary among individuals aged 65 years and older (34%) than among those aged 45–64 years (12%) or 18–44 years (6%). Prevalence varies by gender and ethnicity, with prevalence in women (14%) than in men (12%) and in non-Hispanic black adults (20%) than non-Hispanic Asian adults (14%) or non-Hispanic whites (12%). The NHANES study highlights a markedly low awareness of the disease: approximately 90% of patients with chronic kidney disease (CKD) are unaware of their condition, and even among those with advanced CKD (stage 4), about 30% do not know they have the disease [6]. The annual report of the United States Renal Data System (USRDS) showed that in individuals aged > 60 years, eGFR was below 60 ml/min in more than 35% compared to 0.1% in the 20–39 age group and 2.3% in the 40–59 age group [7]. A systematic study conducted by Hill analyzed the worldwide prevalence of CKD through a systematic review and meta-analysis of 100 observational studies, including over 6.9 million individuals. The results indicated a prevalence of CKD (stages 1–5) of 13.4% (95% CI 11.7–15.1%), with most cases in stage 3 (7.6%) increasing with advancing age, reaching 27.6% in those aged 60 years and 34.3% in those aged 70 years [8]. The CARHES Study (Cardiovascular Risk in Renal Patients of the Health Examination Survey) is an Italian project establishing the prevalence of CKD in the Italian population aged between 35 and 79 years with risk factors for disease progression with the aim of improving the prevention and management of CKD. The prevalence of CKD is resulted to be 6.3%. In particular, the early stages (G1–G2) showed a prevalence of 3.8%, while in the more advanced stages (G3–G5) it was 2.5%. The prevalence of CKD increases significantly with age, rising from 2.7% in the 35–49 age group to 17% in the 70–79 age group, highlighting a role of comorbidities in the genesis and progression of CKD (diabetes mellitus, metabolic syndrome, hypertension, smoking, cardiovascular diseases) [9]. Among the most relevant Italian studies on the epidemiology of CKD is the INCIPE study (Indagine sulla Prevalenza della Insufficienza Cronica Progressiva Renale), conducted in the Veneto Region to estimate the prevalence of CKD in the general population and to identify the main associated risk factors with 3860 adult subjects, randomly selected from residents aged between 40 and 79 years. The diagnosis of CKD was based on eGFR by the MDRD formula and proteinuria assessment. The results showed an overall prevalence of CKD of 12.7% (stages 1–4), in line with data from other countries [10]. An epidemiological study on CKD was the Gubbio Study that retrospectively analyzed 561 patients followed in outpatient care between 2008 and 2012, which showed that more than 50 per cent of the patients were in stage III CKD according to the KDOQI classification, with a prevalence strongly correlated with older age [11]. The decline of eGFR with age is a physiological and not always an indication of pathology, so it is important to distinguish between renal aging (RA) and CKD. Fail to recognize RA can lead to misdiagnosis with clinical, psychological and economic consequences for patients and the National Health Service. Since the Keller equation (GFR = 130—age) determines which is the GFR value expected for age, when eGFR is lower (± 5 ml-min) than the GFR obtained by the Keller equation, this case should be interpreted as CKD. In the elderly, the correct estimation of eGFR avoids diagnostic errors between physiological decline and CKD. Most commonly used formulas, such as the CKD-EPI, tend to overestimate the incidence of CKD in old age, as they do not adequately account for the physiological decline in kidney function. The BIS-1 formula, developed specifically for subjects over 70 years of age, allows a more accurate estimation of eGFR in the geriatric population by incorporating parameters that better reflect age-related changes [12]. A further comparison tool is the Keller formula (GFR = 130—age), which proposes an expected reference value of GFR as a function of chronological age, useful for distinguishing physiological RA from CKD. Integration of BIS-1 and Keller’s reference values may improve diagnostic accuracy, avoiding overdiagnosis of CKD in clinically stable elderly patients. In the context of physiological aging, the assessment of renal function in the elderly requires appropriate tools to distinguish between renal senescence and true pathology. Keller’s formula (GFR = 130—age) represents a useful benchmark for estimating the expected value of glomerular filtrate as a function of age. According to this model, an elderly person can only be considered to have CKD when the eGFR, calculated with CKD-EPI or BIS-1, is at least ± 5 ml/min lower than the expected value according to Keller. This approach allows a more accurate discrimination between physiological reduction of renal function and CKD, avoiding inappropriate diagnosis and treatment in a population with comorbidities and increased clinical frailty. The concept of frailty affects the older population by increasing vulnerability to stress and risk of adverse events [13]. Frailty status can be measured through the Clinical Frailty Scale (CFS), a nine-point scale that classifies the patient according to their functional autonomy and vulnerability to stress (Table 1). Senescent nephropathy (SN) represents the set of morphological and functional renal changes observed in older subjects in the absence of overt renal pathology. With advancing age, there is a physiological decline of eGFR due to glomerular sclerosis, tubular atrophy and interstitial fibrosis, which often leads to eGFR values < 60 ml/min/1.73 m^2^ even in the absence of proteinuria or other urinary abnormalities. Can be confused with CKD, but differs in the absence of significant progression, clinical stability over time and lack of active renal or systemic comorbidities. It is crucial to recognize SN to avoid over-medicalization in older patients with a clinical approach based on overall cardiovascular risk rather than a simple stage-related classification. The distinction between SN and true CKD is particularly important in epidemiological and decision-making contexts, where the physiological decline of eGFR in old age should not automatically lead to pathological diagnoses or unnecessary specialist treatment [14].

**Table 1:** Clinical frailty scale (CFS)

Level	Category	Description
1	Very fit	Robust, active, energetic, motivated; among the fittest for their age
2	Fit	People who have no active disease symptoms but are less fit than category 1
3	Managing well	People whose medical problems are well controlled, but are not regularly active
4	Vulnerable	Although not dependent on others for daily help, often symptoms limit activities
5	Mildly frail	More evident: slowing, and need help with high order IADLs (finances; transportation, heavy housework, medications)
6	Moderately frail	Help is needed with all outside activities and with keeping house; problems with stairs and need help with bathing and might need minimal assistance (cuing, standby) with dressing
7	Severely frail	Completely dependent and approaching the end of life
8	Very severely frail	Completely dependent and approaching the end of life
9	Totally dependent	Completely dependent and approaching the end of life

Another scale to be considered in the large older patient is the Fried Frailty Criteria (FFC) (Table 2) based on 5 parameters: involuntary weight loss, handgrip deficit, reduced walking speed, chronic fatigue, and low level of physical activity. Based on this score, it is possible to classify patients into non-fragile (robust) if no criteria are present, pre-fragile with one or two criteria, fragile with three or more criteria [15].

**Table 2:** Fried Frailty Criteria (FFC)

Criterion	Description
Unintentional weight loss	Weight loss of ≥ 10 lbs (4.5 kg) in the past year
Exhaustion	Self-reported fatigue or inability to get going
Weakness	Low grip strength
Slow walking speed	Walking speed below an established cutoff adjusted for sex and height
Low physical activity	Kcals (or equivalent) of physical activity expenditure per week below an established cutoff
Low physical activity	Kcals (or equivalent) of physical activity expenditure per week below an established cutoff

The prevalence of frailty in patients with CKD ranges from 20 to 50% [16]. Patients with CKD and eGFR < 30 ml/min, have increased disease progression, risk of hospitalization and premature mortality. CKD contributes to the state of frailty through certain risk factors such as chronic inflammatory state and oxidative stress with acceleration of the ageing process, state of malnutrition with reduced protein intake and sarcopenia, reduced physical activity [17]. To date, we do not have a true estimate of CKD in the Abruzzo Region. The Abruzzo Region has a total population as of 01/01/2024 of 1269571. The population over the age of 65 is 325622 (Table 3), i.e. 25.6% of the total population, divided as follows: the 65–74 age group is 159857 (12.6%), the 75–84 age group is 111790 (8.8%), and the 85 + age group is 53975 (4.2%) [1]. The total prevalence of CKD (G3—G5) was 58611 individuals or 4.6% of the total population (Table 4).

**Table 3:** Population Abruzzo over 65 years

			Men		Women	
Abruzzo	1269571		647562		622009	
65-over	325622	25.6%	145092	11.4%	180530	14.2%
65–74	159857	12.6%	75930	6%	83927	6.6%
75–84	111790	8.8%	49925	3.9%	61865	4.9%
85-over	53975	4.2%	19237	1.5%	34738	2.7%

**Table 4:** Cases CKD stage G3-G5 Abruzzo over 65 years

	Total	Incidence on population total
65-over	58611	18%
65–74	8519	2.6%
75–84	17664	5.4%
85-over	32428	9.9%

## Materials and methods

All data were anonymized and aggregated for analysis. The population sample included all individuals aged ≥ 65 years for whom serum creatinine with eGFR values (calculated with CKD-EPI), urinalysis, renal ultrasound, Clinical Frailty Scale (CFS), and Fried Frailty Criteria (FFC) data were available. Exclusion criteria are acute kidney injury, missing or incomplete data, and terminal illness unrelated to renal or frailty evaluation. The aim of the study is to identify the true prevalence of CKD after excluding cases of RA, understood RA as the presence of eGFR (CKD-EPI) equal or higher than Keller (± 5 ml/min), with normal creatinine values, urine examination and ultrasound imaging and no increased risk of mortality [18]. For a better management of the patient with CKD, it is necessary to distinguish between cases of CKD in patients without a fragile state, understood as Robust CKD (RC), and cases of RA understood as SN, Robust Aged Kidney (RAK) and Frailty Aged Kidney (FAK) (Table 5). RC patient is defined as an individual with on one hand CKD, defined as presence of eGFR lower than the expected to his/her age (eGFR value < Keller value), and/or altered urinalysis, and/or abnormal renal imaging, and on the other hand the lack of signs of frailty, able to maintain good autonomy and in particular independent in activities of daily living, a Fried Frailty Criteria < 1 and a CFS ≤ 3 [19]. SN is defined as an individual with on one hand CKD, defined as presence of eGFR lower than the expected to his/her age (eGFR value < Keller value), and/or altered urinalysis, and/or abnormal renal imaging, and on the other hand presence of clinically relevant frailty state (CFS > 3 and FFC > 3)[20]. RAK indicates the presence of eGFR equal or higher than the expected to individual age (eGFR value ≥ Keller value), with normal urinalysis and renal imaging, and on the other hand presence of good functional and cognitive status [21]. FAK indicates the presence of eGFR equal or higher than the expected to individual age (eGFR value ≥ Keller value), with a state of physical and/or cognitive frailty, according to validated criteria (e.g. Clinical Frailty Scale ≥ 4, or ≥ 1 criterion according to Fried et al.) [22]. The analysis of the over-65 population living in Abruzzo (*n* = 325,622) allowed the identification and classification of subjects affected by impaired renal function according to four specific categories: RC, SN, RAK and FAK. The classification was based on the combination of laboratory parameters (eGFR, creatinine, urine examination), ultrasound imaging and indicators of frailty (CFS, Fried Frailty Criteria). The reclassification of patients aged 65 years and older using Keller’s formula (GFR = 130 − age) allows for a more age-appropriate interpretation of renal function decline. By estimating the expected GFR based on age, it becomes possible to distinguish RA from true CKD. The graphical representation highlights how a substantial proportion of older patients fall within GFR values that are consistent with normal aging, particularly in the categories of RAK and FAK. This distinction is critical, as it prevents overdiagnosis of CKD in elderly individuals whose reduced GFR aligns with age-related norms rather than pathology. Integrating Keller’s reference values into epidemiological analyses may improve diagnostic accuracy and promote more appropriate clinical decision-making, avoiding unnecessary interventions in cases of benign senescent nephropathy. This is a retrospective observational study carried out from January 2024 to May 2025 on the population aged ≥ 65 years residing in the Abruzzo Region. The study aimed to assess the prevalence and characteristics of CKD, with attention to distinguishing CKD from RA using age-adapted formulas and frailty assessment tools. Descriptive statistics were used to calculate frequencies, percentages, medians, and interquartile ranges for demographic and clinical variables. Patients were stratified into four renal aging categories based on combinations of eGFR (vs Keller reference), proteinuria, imaging findings, and frailty status (CFS and FFC). Cross-tabulations and graphical representations were used to visualize category distribution. Statistical software (e.g., Excel, SPSS) was used for data analysis. Due to the descriptive nature of the study and heterogeneity of available data, no inferential statistical tests or multivariate models were performed. The study was conducted in accordance with the ethical principles outlined in the Declaration of Helsinki. All patient data were anonymized at the point of collection, and no personal identifiers were used in analysis or dissemination. Since this study relied on routinely collected health data and posed no additional risks to patients, formal ethical approval was waived by the regional health authority.

**Table 5:** Classification of Renal Categories in the Elderly

	Robust CKD	Senescent nephropathy	Robust aged kidney	Frailty aged kidney
GFR	eGFR < Keller (± 5 ml/min)	eGFR < Keller (± 5 ml/min)	eGFR ≥ Keller (± 5 ml/min)	eGFR ≥ Keller (± 5 ml/min)
Proteinuria	Variable	Variable	No	No
Frailty	Absent	Present	Absent	Present
Functional status	Independent	Reduced	Independent	Reduced
Comorbidity	Mild	Moderate or severe	Mild	Moderate or severe

## Results

A total of 58,611 patients with CKD stage G3–G5 (prevalence 18%) were identified in patients ≥ 65 years (Table 6).

**Table 6:** Abruzzo’s patients with CKD stage G3-G5

age	G3a	G3b	G4	G5	Total
65–74 years	6142 (72.1%)	1835 (21.5%)	420 (4.9%)	122 (1.4%)	8519
75–84 years	7773 (44%)	7825 (44.3%)	1713 (9.7%)	353 (2%)	17664
85 years-over	6674 (20.6%)	8230 (25.4%)	13743 (42.4%)	3781 (11.6%)	32428

The analysis of data on the population over 65 resident in the Abruzzo Region has highlighted an important discrepancy between the estimated prevalence of CKD according to the traditional criteria and that recalculated on the basis of age. The values obtained by means of the CKD-EPI formula, the overall prevalence of CKD in the G3–G5 stages increases significantly with age: it goes from 8519 cases in the 65–74 age group, to 17664 cases in the 75–84 age group, up to 32428 cases in the over-85 age group (Table 6). However, this approach risks overestimating the true incidence of CKD in elderly patients, confusing the physiological decline in kidney function with a pathological condition. By applying Keller’s formula, which takes into account the physiological age-related decrease in glomerular filtrate, the number of truly pathological cases of CKD is substantially reduced. In the 65–74 age group, only 27.9% of the cases initially classified as CKD have an eGFR lower than the expected value for age; in the 75–84 and ≥ 85 age groups, the percentage of true CKD cases is 56 and 54%, respectively (Table 7–8). Based on the results obtained, four groups were identified: (1) robust CKD (CKD without frailty); (2) senescent nephropathy (SN) understood as the presence of CKD with frailty; (3) robust Aged Kidney (renal ageing without frailty); (4) frailty aged kidney (renal ageing with frailty). The reclassification of patients aged 65 years and older using Keller’s formula (GFR = 130 − age) allows for a more age-appropriate interpretation of renal function decline. By estimating the expected GFR based solely on age, it becomes possible to distinguish physiological renal aging from true chronic kidney disease (CKD). The graphical representation highlights how a substantial proportion of older patients fall within GFR values that are consistent with normal aging, particularly in the categories of Robust Aged Kidney (RAK) and Frailty Aged Kidney (FAK). This distinction is critical, as it prevents overdiagnosis of CKD in elderly individuals whose reduced GFR aligns with age-related norms rather than pathology. Integrating Keller’s reference values into epidemiological analyses may improve diagnostic accuracy and promote more appropriate clinical decision-making, avoiding unnecessary interventions in cases of benign senescent nephropathy. In detail, the Robust CKD category, which identifies individuals with CKD in the absence of clinical frailty, is represented by 1115 individuals (1.9%) in the 65–74 age group, 1856 (3.16%) between 75 and 84 years, and 3323 (5.66%) over 85 years. Senescent Nephropathy (SN), defined by the presence of CKD in frail subjects but with preserved laboratory and instrumental parameters, was particularly prevalent in the older age group, with 1147 patients (1.95%) aged 65–74 years, 7274 (12.41%) aged 75–84 years and 12,894 subjects (22%) over 86 years. The Robust Aged Kidney category, which represents the physiological decline in kidney function in elderly subjects without frailty and with normal parameters, was observed in 4791 patients (8.17%) aged 65–74 years, in 2635 patients aged 75–84 years (4.49%) and in 4835 patients aged > 85 years (8.25%). Finally, Frailty Aged Kidney, indicative of a functional renal impairment associated with a state of clinical frailty and altered parameters, showed an increasing distribution with age: 1466 patients (2.5%) in the 65–74 age group, 5899 patients (10%) between 75 and 84 years, and as many as 11376 patients (19.4%) in the over 86 age group. These data show that more than 50 per cent of patients over the age of 65 with CKD in Abruzzo fall into one of the two clinically fragile categories (SN and Frailty Aged Kidney), underlining the importance of a multidimensional approach in the assessment and management of CKD in the geriatric population. The analysis of the data concerning the over-65 population in the Abruzzo Region showed a substantial difference between the conventional estimate of the prevalence of CKD (Table 7) and that recalculated according to Keller’s formula (Table 9). In particular, in the 65–74 age group, the number of patients with stages G3–G5 was 8519, but only 2377 (27.9%) had a glomerular filtrate actually below the expected age threshold. In the 75–84 age group, the number of CKD cases fell from 17664 to 9891 (56%), while among the over-85 s, the true CKD cases were 17524 out of 32428 (54%), showing that more than 40% of the cases classified as CKD according to CKD-EPI actually fall within the range of age-related physiological decline. Further confirmation of this overestimation emerges when comparing the distribution of the four clinical phenotypes (Table 10) with their recalibration according to Keller and BIS-1 (Table 11). In the initial classification, patients classified as ‘Robust CKD’ totalled 6294, whereas in the corrected estimate the number is reduced to 3216. Similarly, the number of patients with ‘Senescent Nephropathy’ also drops from 25315 to 12214. On the other hand, the number of subjects with ‘Robust Aged Kidney’ and ‘Frailty Aged Kidney’ redistributes significantly, better reflecting the physiological nature of the decline in renal function in the absence of instrumental changes or marked clinical frailty.

**Table 7:** Real CKD estimate calculation according to Keller

Age	CKD EPI ≥ Keller	CKD EPI < Keller	% real CKD
65–74 years	2377	8519	27.9%
75–84 years	9891	17664	56.0%
85 years – over	17524	32428	54.0%

**Table 8:**
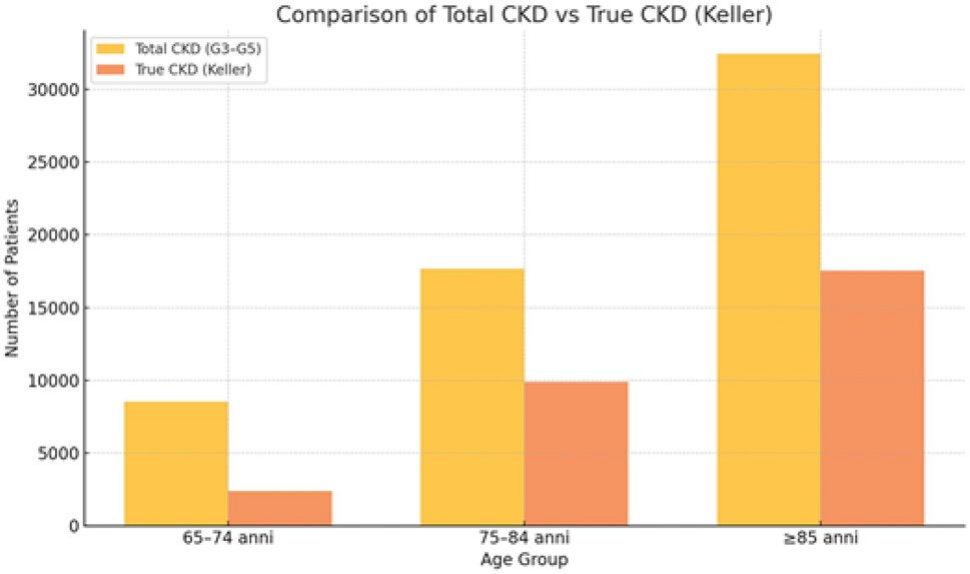
Comparison between Total CKD – EPI vs CKD ≥ Keller

**Table 9:** Estimated CKD Categories by Age Group

Category	Age group	Estimated patients
Robust CKD	65–74	1115 (1.9%)
Robust CKD	75–84	1856 (3.16%)
Robust CKD	85 +	3323 (5.66%)
Senescent nephropathy (SN)	65–74	1147 (1.95%)
Senescent nephropathy (SN)	75–84	7274 (12.41%)
Senescent nephropathy (SN)	85 +	12,894 (22%)
Robust aged kidney	65–74	4791 (8.17%)
Robust aged kidney	75–84	2635 (4.49%)
Robust aged kidney	85 +	4835 (8.25%)
Frailty aged kidney	65–74	1466 (2.5%)
Frailty aged kidney	75–84	5899 (10%)
Frailty aged kidney	85 +	11376 (19.4)

**Table 10:** Estimated CKD Categories by Keller formula e BIS-1

Category	Age group	Estimated patients
Robust CKD	65–74	625 (2.1%)
Robust CKD	75–84	864 (2.9%)
Robust CKD	85 +	1727 (5.8%)
Senescent nephropathy (SN)	65–74	596 (2%)
Senescent nephropathy (SN)	75–84	4468 (15%)
Senescent nephropathy (SN)	85 +	7150 (24%)
Robust aged kidney	65–74	214 (0.7%)
Robust aged kidney	75–84	1521 (5.1%)
Robust aged kidney	85 +	5243 (17.6%)
Frailty aged kidney	65–74	942 (3.1%)
Frailty aged kidney	75–84	3038 (10.2%)
Frailty aged kidney	85 +	3404 (11.4)

**Table 11:**
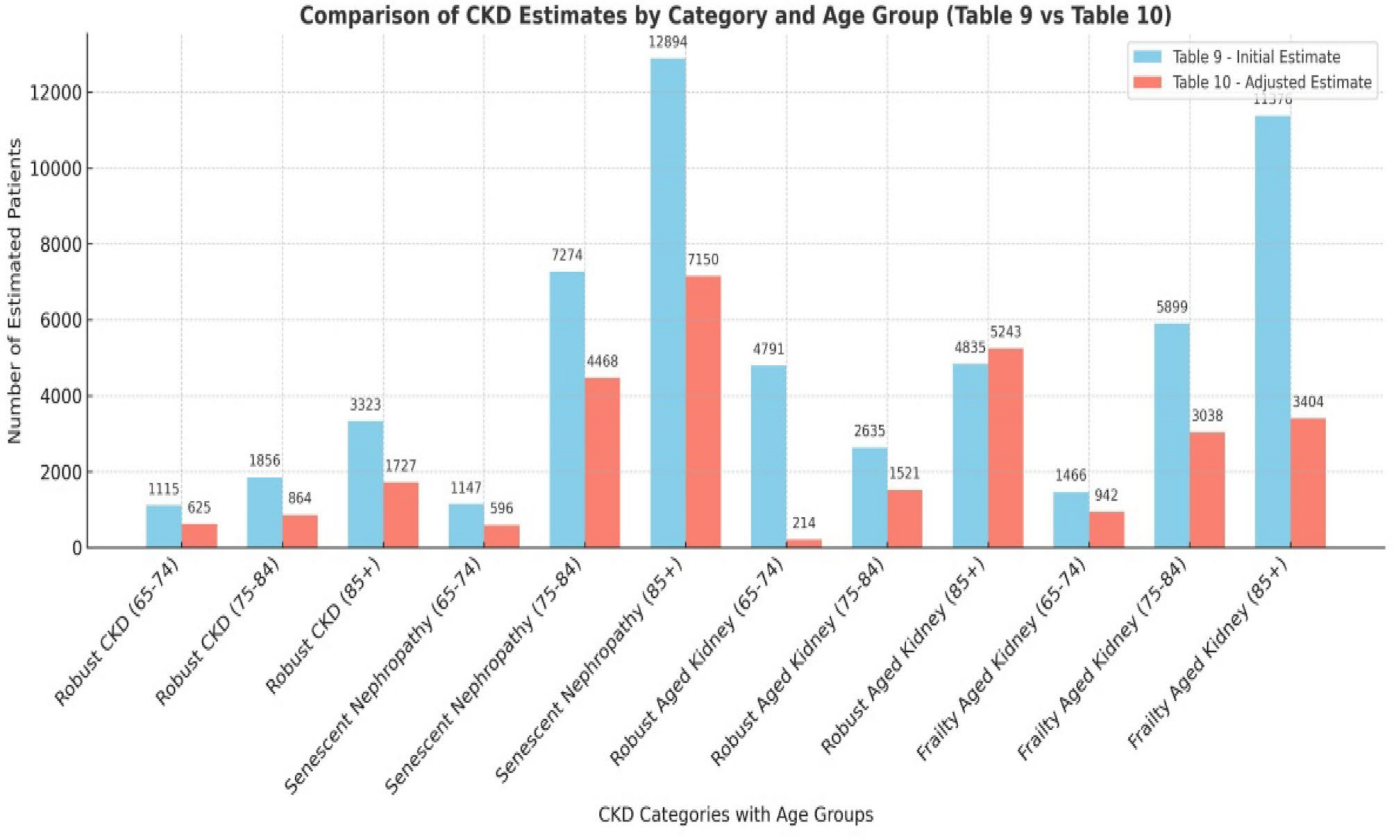
comparison of CKD Categories

The distribution of patients in the different categories shows an increasing prevalence of the frail and senescent forms with advancing age. In detail, the Robust CKD category, which identifies individuals with CKD in the absence of clinical frailty, is represented by 1115 individuals (1.9%) in the 65–74 age group, 1856 (3.16%) between 75 and 84 years, and 3323 (5.66%) over 85 years. Senescent Nephropathy (SN), defined by the presence of CKD in frail subjects but with preserved laboratory and instrumental parameters, was particularly prevalent in the older age group, with 1147 patients (1.95%) aged 65–74 years, 7274 (12.41%) aged 75–84 years and 12,894 subjects (22%) over 86 years. The Robust Aged Kidney category, which represents the physiological decline in kidney function in elderly subjects without frailty and with normal parameters, was observed in 4791 patients (8.17%) aged 65–74 years, in 2635 patients aged 75–84 years (4.49%) and in 4835 patients aged > 85 years (8.25%). Finally, Frailty Aged Kidney, indicative of a functional renal impairment associated with a state of clinical frailty and altered parameters, showed an increasing distribution with age: 1466 patients (2.5%) in the 65–74 age group, 5899 patients (10%) between 75 and 84 years, and as many as 11376 patients (19.4%) in the over 86 age group. These data show that more than 50 per cent of patients over the age of 65 with CKD in Abruzzo fall into one of the two clinically fragile categories (SN and Frailty Aged Kidney), underlining the importance of a multidimensional approach in the assessment and management of CKD in the geriatric population. More than 40% of the cases classified as CKD according to CKD-EPI actually fall within the range of age-related physiological decline.

## Discussion

Based on the results obtained, four groups were identified robust CKD (CKD without frailty), senescent nephropathy (SN) understood as the presence of CKD with frailty, robust Aged Kidney (renal ageing without frailty), frailty aged kidney (renal ageing with frailty). The reclassification of patients aged 65 years and older using Keller’s formula (GFR = 130 − age) allows for a more age-appropriate interpretation of renal function decline. By estimating the expected GFR based solely on age, it becomes possible to distinguish physiological renal aging from true chronic kidney disease (CKD). The graphical (Table 12) representation highlights how a substantial proportion of older patients fall within GFR values that are consistent with normal aging, particularly in the categories of Robust Aged Kidney (RAK) and Frailty Aged Kidney (FAK). This distinction is critical, as it prevents overdiagnosis of CKD in elderly individuals whose reduced GFR aligns with age-related norms rather than pathology. Integrating Keller’s reference values into epidemiological analyses may improve diagnostic accuracy and promote more appropriate clinical decision-making, avoiding unnecessary interventions in cases of benign senescent nephropathy. Further confirmation of this overestimation emerges when comparing the distribution of the four clinical phenotypes with their recalibration according to Keller and BIS-1 (Table 11). In the initial classification, patients classified as ‘Robust CKD’ totalled 6294, whereas in the corrected estimate the number is reduced to 3216. Similarly, the number of patients with ‘Senescent Nephropathy’ also drops from 25,315 to 12,214. On the other hand, the number of subjects with ‘Robust Aged Kidney’ and ‘Frailty Aged Kidney’ redistributes significantly, better reflecting the physiological nature of the decline in renal function in the absence of instrumental changes or marked clinical frailty. These data underline the importance of a personalised approach in the diagnosis of CKD in the geriatric patient, integrating age-adjusted predictive formula (such as Keller) and clinical frailty indicators, in order to avoid the risk of overdiagnosis and unindicated treatment. Table 12 gives a reduction in patients classified as CKD with adjusted criteria, and an increase in physiological ageing categories, emphasizing the importance of age-adjusted evaluation in elderly populations. The current Kidney Disease Outcomes Quality Initiative (KDOQI) classification of 2012 considers chronic kidney disease (CKD) based on eGFR and albuminuria. Although age plays a role in the calculation of eGFR, this classification does not consider corrective factors based on the patient’s age, with the risk of overestimating the incidence of CKD in the elderly population (over 65 years of age). From the age of 40 years according to Keller’s formula eGFR decreases by approximately 1 ml/min per year [23]. A valid and accurate alternative for estimating GFR in the elderly population is the European Kidney Function Consortium equation (EKFC) [24]; however, in the present study, the Keller formula (age-expected GFR = 130—age) was chosen for its greater ease of use, practical applicability, and immediate interpretability. This approach proved particularly effective in collaboration with general practitioners, for whom practical, quick, and easily integrable tools are essential in daily clinical practice. The use of the Keller formula also promotes more effective communication between specialists and primary care physicians in the management of elderly patients, contributing to a reduction in the risk of CKD overdiagnosis in older adults and its potential negative clinical consequences [23]. RA is a natural phenomenon of declining kidney function that does not necessarily indicate a disease state. The current classification risks creating overdiagnosis of CKD with a negative impact both psychologically (anxiety, depression) and increased healthcare costs with inappropriate therapy prescriptions, such as hypoproteic diets with the risk of developing sarcopenia and the risk of unnecessary treatment costs that could also prove harmful [25]. In the classification of CKD, it is necessary to avoid misdiagnosis of CKD in the elderly population and to consider in the KDIGO classification (Table 12  a grey area in the categories G3a/A1 in subjects 70–85 years called the ‘alpha (α)’ zone and G3b/A1 in subjects 86–100 years called the ‘omega (ω)’ zone [13].

**Table 12:** Classification CKD in elderly population

GFR	A1 (< 30 mg/g)	A2 (30–300 mg/g)	A3 (> 300 mg/g)
G1 (≥ 90)	Low risk	Moderate risk	High risk
G2 (60–89)	Low risk	Moderate risk	High risk
G3a (45–59)	Alpha (α)	High risk	Very high risk
G3b (30–44)	Omega (ω)	Very high risk	Very high risk
G4 (15–29)	Very high risk	Very high risk	Very high risk
G5 (< 15)	Very high risk	Very high risk	Very high risk

G stage CKD—A Albuminuria

The relationship between frailty and CKD was considered with the concept of senescent nephropathy (SN) describing the condition of frail patients with CKD. Frailty characterized by reduced physical activity and vulnerability to stress, is highly prevalent in the elderly population and has a significant impact on the evolution of CKD and quality of life. This classification is useful for personalising treatment and estimating the prognosis for each specific patient, such as access to dedicated nephrology outpatient clinics and for planning intensive treatments such as preparation for haemodialysis treatment. The progressive ageing of the population has led to a significant increase in CKD in patients over the age of five with a risk of needing dialysis initiation; today, around 40% of new dialysis admissions each year are over 80 years old [26]. These patients are often frail as they are elderly or very old with multiple comorbidities (heart disease, diabetes, vasculopathy) fragility that is accentuated in some cases as the outcome of acute events or complex interventional procedures considered incompatible with such patients until a few years ago. Patients with ESRD present a high frailty with higher rates of hospitalisation and death than patients of the same age without MRC [27]. In elderly patients, the start of dialysis is associated with rapid loss of functional autonomy, with frequent hospitalisations and reduced survival. The study of “disease trajectories” shows that advanced CKD not yet on dialysis has a worse prognostic course than some solid tumours [28]. This is supported by the document “Palliative care in patients with advanced MRC” [29] shared between SIN (Italian Society of Nephrology) and SICP (Italian Society of Palliative Care) which highlighted as unfavourable prognostic factors age over 75 years, the type and severity of comorbidities, malnutrition, severe cognitive impairment, reduced functional autonomy and frequent hospitalisations. With the significant increase in frailty, there has also been a growing focus on a comprehensive assessment of the individual patient before proposing dialysis treatment, especially when an inauspicious short-term prognosis can be assumed. All this is exacerbated in frail elderly patients who, with the start of dialysis, risk having their quality of life compromised with the remaining time of their life spent in hospital due to the occurrence of complications linked to both the invasive manoeuvres preparatory to the start of dialysis therapy (central venous dialysis catheter placement or peritoneal catheter FAV packaging) and dialysis therapy with the reflection that starting elderly patients with severe comorbidities on dialysis is tantamount to postponing death rather than saving life. It is necessary to overcome the uremia-dialysis paradigm in elderly patients and to consider a maximal conservative therapy called non-aggressive renal therapy characterised by a personalised medical therapy focused on the individual patient’s needs to achieve symptom control and the greatest well-being [30]. For a successful outcome, it is necessary that the pathway is shared both with the patient and especially with family members. Advance Care Planning (ACP), a shared care pathway involving the patient, the family and the general practitioner (GP) in a chronicity management programme. ACP envisaged by Law 219 of 22/12/17 in Article 4 in the nephrology field provides for: total active care of the patient and family, management of physical pain and the psychological, spiritual and cultural implications arising from the state of illness; professional support in deciding whether to start or suspend dialysis treatment; involvement in the discussion on end-of-life care to have a dignified death; choice with the family and patient of the place of death; structuring of the entire palliative care pathway both in hospital and at home [31]. Even when a decline in eGFR reflects age-related physiological changes rather than overt nephropathy, it may still carry significant clinical consequences. A reduced eGFR has been associated with elevated cardiovascular risk and requires careful consideration when adjusting drug dosages [32]. eGFR remains a key parameter for pharmacokinetic decisions, irrespective of whether the decline is pathological or age-related. Therefore, even in cases where the Keller formula or other age-calibrated tools suggest preserved physiological aging, clinicians must remain alert to the broader implications of reduced kidney function. This further supports the need for an integrative approach combining age-adapted GFR estimation, frailty assessment, and individualized cardiovascular and therapeutic risk profiling [33].

## Conclusions

The present study highlighted how the ageing of the population, a demographic phenomenon that is particularly marked in the Abruzzo Region, represents an important epidemiological determinant in the increase in the prevalence of CKD. However, the association between advanced age and reduced renal function is not always an expression of a pathological condition: the physiological decline in glomerular filtrate must be distinguished from pathological forms of CKD to avoid improper diagnosis and consequent inappropriate treatment. The integration of laboratory data (eGFR, creatinine, urine examination), ultrasound imaging and fragility assessment tools (Clinical Frailty Scale, Fried Frailty Criteria) has made it possible to propose a more refined and clinically relevant classification of CKD in subjects over 65 years of age, dividing them into four categories: Robust CKD, Senescent Nephropathy, Robust Aged Kidney and Frailty Aged Kidney. This distinction proved crucial for a correct interpretation of epidemiological data and better planning of clinical care. The data collected showed that over 50% of patients over 65 years of age suffering from CKD fall into the clinically frail categories (SN and FAK), underlining the need for a multidimensional geriatric approach in the care of these subjects. In particular, the relevance of frailty as an independent prognostic factor for mortality, hospitalisation and reduced functional autonomy in patients with CKD emerged. In light of this evidence, a rethinking of the diagnostic and management criteria for CKD in the elderly patient is proposed. The use of static classifications based solely on eGFR runs the risk of overestimating the prevalence of CKD in the geriatric population, generating a potential over-medication. Instead, the inclusion of frailty parameters and the identification of conditions such as senescent nephropathy or robust renal ageing allow greater personalisation of therapeutic decisions, avoiding invasive treatments that are not indicated. Finally, the high prevalence of frail patients with CKD raises the urgency of shared care pathways focused on realistic goals of care, including conservative management models for advanced CKD. In this context, Advance Care Planning and collaboration between nephrologists, geriatricians, general practitioners and families assume a central role to ensure a dignified quality of life for the elderly patient, respecting his preferences and the limitations imposed by frailty.

## Data Availability

No datasets were generated or analysed during the current study.
